# Endorsement and embodiment of cautiousness-related age stereotypes

**DOI:** 10.3389/fpsyg.2023.1091763

**Published:** 2023-01-26

**Authors:** Tingting Huang, Klaus Rothermund

**Affiliations:** Institute of Psychology, Friedrich Schiller University Jena, Jena, Germany

**Keywords:** implicit age stereotypes, stereotype endorsement, self-stereotyping, stereotype embodiment, cautious behavior

## Abstract

Endorsement of implicit age stereotypes was assessed with the propositional evaluation paradigm (PEP) in a high-powered, preregistered study, comprising samples of young (*n* = 89) and older (*n* = 125) adults. To investigate whether implicit age stereotypes shape the behavior *via* self-stereotyping (“embodiment”), we examined whether implicit endorsement of the belief of older (young) people being cautious (reckless) predicts older (young) individuals’ spontaneous behavior in a speeded response time task. In both age groups, we found significant implicit endorsement effects of age stereotypical beliefs. However, implicit endorsement effects of the cautiousness-related age stereotypes were unrelated to our indicators of spontaneous cautious/reckless behavior in the speeded RT task (as assessed with the parameter *a* of a diffusion model analysis) for both age groups. The same pattern of results (endorsement of age stereotypic beliefs but no relation with behavioral indicators) was found for explicit measures of age stereotypes. Replicating previous findings, implicit and explicit measures of cautiousness-related age stereotypes were uncorrelated. In sum, our findings provide evidence for the implicit and explicit endorsement of cautiousness-related stereotypical beliefs about old and young people; individual differences in belief endorsement, however, did not predict differences in spontaneous cautiousness-related behavior in a speeded RT task.

## Introduction

Stereotypes have been shown to shape development *via* processes of self-stereotyping (Smith and Henry, 1996; Lun et al., 2009; Casper and Rothermund, 2012). In the domain of age stereotypes, the internalization process begins at early childhood and become reinforced in adulthood (Levy, 2003). As reaching an old age, individuals at first tend to deny that they are old and to resist applying negative age stereotypes to themselves. It has been found that the more negative the aging stereotypes are, the more resistance there would be to identifying with the old (Levy and Banaji, 2002; Levy, 2009). However, as encountering with a plethora of old-age cues, older individuals are prompted to identify with their cohort and the negative aging stereotypes now become self-relevant (Hummert et al., 2002; Levy, 2003; Rothermund and Brandtstädter, 2003; Kornadt and Rothermund, 2012; Kornadt et al., 2017; Voss et al., 2017). In support of the determinant role of self-categorization in this approach, for example, Kornadt and Rothermund (2012) found that age stereotypes become internalized into self-concept only when older individuals categorize themselves as being old. After the transition of age stereotypes to self-relevance, the chronic activation of these stereotypes that include many negative expectations for future functioning then influence the behavior, motivation, cognition, and even physiology of older people *via* embodiment (Levy, 2009; Kornadt and Rothermund, 2012; Kornadt et al., 2017; Voss et al., 2017; Wurm et al., 2017; Rothermund et al., 2021; Rothermund, 2022).

Studies on the consequences of age-related self-stereotyping among older people have mostly relied on an explicit assessment of age stereotypes *via* self-report. Given that stereotypes often reflect automatic ascriptions of attributes to social categories that people are sometimes incapable of reporting or unwilling to admit (Greenwald and Banaji, 1995), it is promising to also investigate effects of implicit age stereotypes on older people’s behavior. To date, however, only few investigated the effects of implicit age stereotypes on behavior, and these studies did not yet provide convincing evidence for processes of embodiment (implicit-criterion correlations ranging between −0.21 and 0.03; Kurdi et al., 2019).

### Implicit measure of stereotypical beliefs

One possible explanation for the low predictive validity of implicit measures of age stereotypes is that these measures typically aim at assessing simple associations between the category “old” and evaluative attributes. These measures of associations, however, are notoriously unreliable and ambiguous with regard to their interpretation (Meissner et al., 2019; Rothermund et al., 2020; Sherman and Klein, 2021). On the one hand, associations do not have a clear meaning (*cf.* De Houwer et al., 2015). For instance, an association between “old” and “cautious” can reflect the proposition that “old people are cautious,” but can also reflect the prescriptive belief that “old people should be cautious” (e.g., de Paula Couto et al., 2022a; see also de Paula Couto et al., 2022b). On the other hand, simple associative relations between the category “old” and specific attributes (e.g., old—slow, old—experienced) have been shown to be extremely fragile and unreliable, with simple category primes being unable to elicit an activation of specific attributes (Casper et al., 2011; Huang and Rothermund, in press; Müller and Rothermund, 2014; see Kidder et al., 2018, for a review). The reason for this lack of simple stereotype activation effects in category-attribute priming is the context-dependent nature of age stereotypes (Casper et al., 2010, 2011): It thus takes a combination of the category “old” and a specific context to activate a specific age-stereotypic attribute. For instance, in order to activate the attribute “slow,” the category “old” has to be combined with a matching context, like “walking across the street”; combining it with no or a mismatching context like “watering the flowers” will not produce activation for “slow” (Casper et al., 2011; Huang and Rothermund, 2022; see also Wigboldus et al., 2003).

A possible solution for these problems is provided by recent suggestions of “relational” indirect measures (Barnes-Holmes et al., 2010; De Houwer et al., 2015) that allow for an assessment of propositions rather than simple associations. These measures allow researchers to (a) specify the exact meaning of the relation between category and the stereotypic attribute, and (b) to incorporate a combination of category and context information into the proposition the endorsement of which is to be assessed. A promising new paradigm of this type that is optimally suited for an implicit assessment of context-dependent age stereotypes is the propositional evaluation paradigm (PEP; Müller and Rothermund, 2019; see also Cummins and De Houwer, 2021; de Paula Couto and Rothermund, 2022). In each trial of the PEP, a statement reflecting a specific belief is presented (e.g., “When driving a car, young people are reckless” or “When driving a car, old people are reckless”). After that, either the target word “True” or the target word “False” is presented, and participants have to indicate the identity of the target word that has been shown by pressing one of two corresponding keys. Importantly, participants give their responses only depending on the target words, regardless of whether they consider the previously shown sentence prime to be true or false. The extent to which a statement is implicitly endorsed is measured by the reaction time difference for responses to “True” and “False” targets, with faster responses to “True” targets indicating endorsement of the statement (see also Wiswede et al., 2013).

### Spontaneous cautious behavior

Another possible reason for the lack of evidence regarding the relation between implicit age stereotypes and corresponding behaviors is that the nature of the behaviors that were measured in previous studies may not have been optimally suited to detect such an influence. In line with dual process models (e.g., RIM, Strack and Deutsch, 2004), a great deal of research on the predictive validity of implicit and explicit attitudes revealed a double-dissociation pattern that implicit measures uniquely predict spontaneous or impulsive behaviors and the controlled actions that are based on conscious reflection are uniquely predicted by explicit measures (e.g., Asendorpf et al., 2002; Dovidio et al., 2002; Egloff and Schmukle, 2002; Perugini, 2005). Previous studies investigating the link between implicit age stereotypes and behavior (e.g., Lindner, 2011; Ruiz et al., 2015), however, have assessed self-reported behaviors or behavioral intentions, which may reflect controlled rather than spontaneous aspects of behavior. We tried to overcome this problem by employing a more indirect indicator of stereotypical behavior in our study that is based on the speed of responding, which may reflect a more spontaneous aspect of behavior that is not controlled by conscious reflection.

The current study specifically focused on the dimension of reckless vs. cautious behavior. It has been a persistent notion among lay persons and psychologists that increasing age leads to increasing cautiousness or conservatism, manifesting itself in age group differences in various behaviors, such as driving, decision making, accuracy-based cognitive task performance, and so on (Okun, 1976). However, cautious or reckless behavior as age stereotypical behaviors, have been neglected by research on stereotype-behavior relations: According to a meta-analysis of the behavioral effects of age-based stereotypes, only two out of 37 studies involved stereotypical beliefs about older people being overly cautious by investigating the stereotypes of older adults’ poor driving ability and their impact on driving performance (Lamont et al., 2015). Thus to know more about the influence of age stereotypes on one’s cautious behavior, we investigated whether one’s endorsement of in-group age stereotypes about cautiousness or recklessness would impact their own behavior. By focusing on age stereotypical beliefs, our study also helps us to understand more about the origins of cautious or reckless behavior.

Considering that the implicitly endorsed stereotypes should allow for a unique prediction of spontaneous cautious behaviors, we computed the *a* parameter of the diffusion model (i.e., the individual threshold of evidence accumulation that is needed to make a behavioral decision, Voss et al., 2004) in a speeded response time task for each participant, which served as our criterion for reckless vs. cautious behavior. The threshold reflects an indicator of the individual speed-accuracy tradeoff that is independent of individual differences in performance quality (e.g., overall response speed and accuracy). The *a* parameter has previously been shown to capture age group differences in performance in speeded response time tasks (e.g., Ratcliff et al., 2000, 2001), and has been established as a highly sensitive indicator of an individual’s mode of functioning during speeded tasks (Voss et al., 2004). Although cautious vs. reckless behavior is partly under voluntary control and can result from conscious decisions to be careful or daring, a large part of the variance in speed-accuracy tradeoffs results from spontaneous adaptations of behavior to task demands (e.g., speed-accuracy tradeoffs are automatically adapted to task difficulty or error feedback; Botvinick et al., 2001; Dutilh et al., 2012; Dambacher and Hübner, 2013). It thus seems promising to investigate whether parts of this adaptive behavioral regulation are automatically influenced by implicit in-group stereotypes of being cautious or daring.

### The current study

Our aims in this study are to (1) assess young and older adults’ endorsement of implicit age stereotypical beliefs in relevant contexts; and to (2) investigate whether the endorsement of implicit age stereotypes of old (young) people being cautious (reckless) predicts older (young) adults’ cautious behavior. To compare the endorsement and embodiment of implicit age stereotypes with explicit age stereotypes, we also assessed explicit endorsement of age stereotypes for the same statements that were presented in the PEP task. Additionally, we also measured the self-reported cautiousness during the PEP task as another behavioral criterion.

Regarding the endorsement of age stereotypes among young and older adults, we expect to see endorsement of age stereotypic statements as assessed explicitly (ratings) or implicitly (PEP) in relevant contexts (e.g., “When driving a car, young people are reckless.”) rather than in irrelevant contexts (e.g., “When watering flowers at home, young people are reckless.”).

Regarding effects of self-stereotyping and embodiment, we hypothesize that among older adults, individual differences in the implicit endorsement of the stereotype that older adults are cautious become internalized and thus should shape their behavior, that is, they should predict individual differences in speed-accuracy tradeoffs during a speeded classification task. Old—but not young—individuals who more strongly endorse this belief should have a higher *a* parameter, indicating that they adjust their performance toward accuracy by accumulating more information before making a behavioral decision. Similarly, we predicted an influence of individual differences in the implicit endorsement of in-group stereotypes about young people being daring to influence the accuracy motivation of young participants. Young—but not old—participants who more strongly endorse the in-group stereotype that young people are daring should have a lower *a* parameter, indicating that their performance is geared toward giving speeded responses at the cost of incurring relatively more errors. For explicitly endorsed age stereotypes, we made similar age group specific predictions for self-reported cautiousness as the outcome.

## Materials and methods

### Sample

Within each age group, the sample size required to detect a medium-sized effect regarding the incremental predictive validity of the implicit measure (*R*^2^ increase: *f*^2^ = 0.1) with sufficient power (1−ß = 0.8) was 81. We aimed at collecting data for 90 young (18–30 years old) and 90 older (older than 65 years old) participants to allow for the possibility that some participants might drop out of the analyses due to our exclusion criteria. *Via* Respondi, we collected data for 91 young (79.12% female, *M_age_* = 25.58 years, *SD* = 3.58) and 127 older participants^1^ (28.35% female, *M_age_* = 71.69 years, *SD* = 4.25) in an online study. After completing the experiment, all participants were paid for their participation. The study was pre-registered on PsychArchives.^2^ All the data are also available on PsychArchives.^3^

### Materials

Thirty-two stereotypic statements for the PEP and for the explicit rating task were created based on eight stereotypic attributes, four of which were stereotypic for young people (e.g., strong, fast, naive, and reckless) and another four were stereotypic for old people (e.g., cautious, experienced, helpless, and slow). For each of the eight attributes, four statements were created by composing of a phrase describing a specific activity or situation that was either relevant or irrelevant for the respective attribute, and ascribing this attribute in this situation to either the matching or mismatching age category (e.g., reckless—“When driving a car/When watering flowers at home, young/old people are reckless”; cautious—“When protecting themselves against the Corona virus/When buying fruits in the supermarket, old/young people are cautious”).

For the catch trials in the PEP task, in addition to the 32 stereotypic statements that were used as sentence primes in the PEP, we also created 32 non-stereotypic statements based on eight non-stereotypic attributes (e.g., “When earning millions each year/When painting the walls at home, young/old people are successful”).

### Procedure

**Figure 1:**
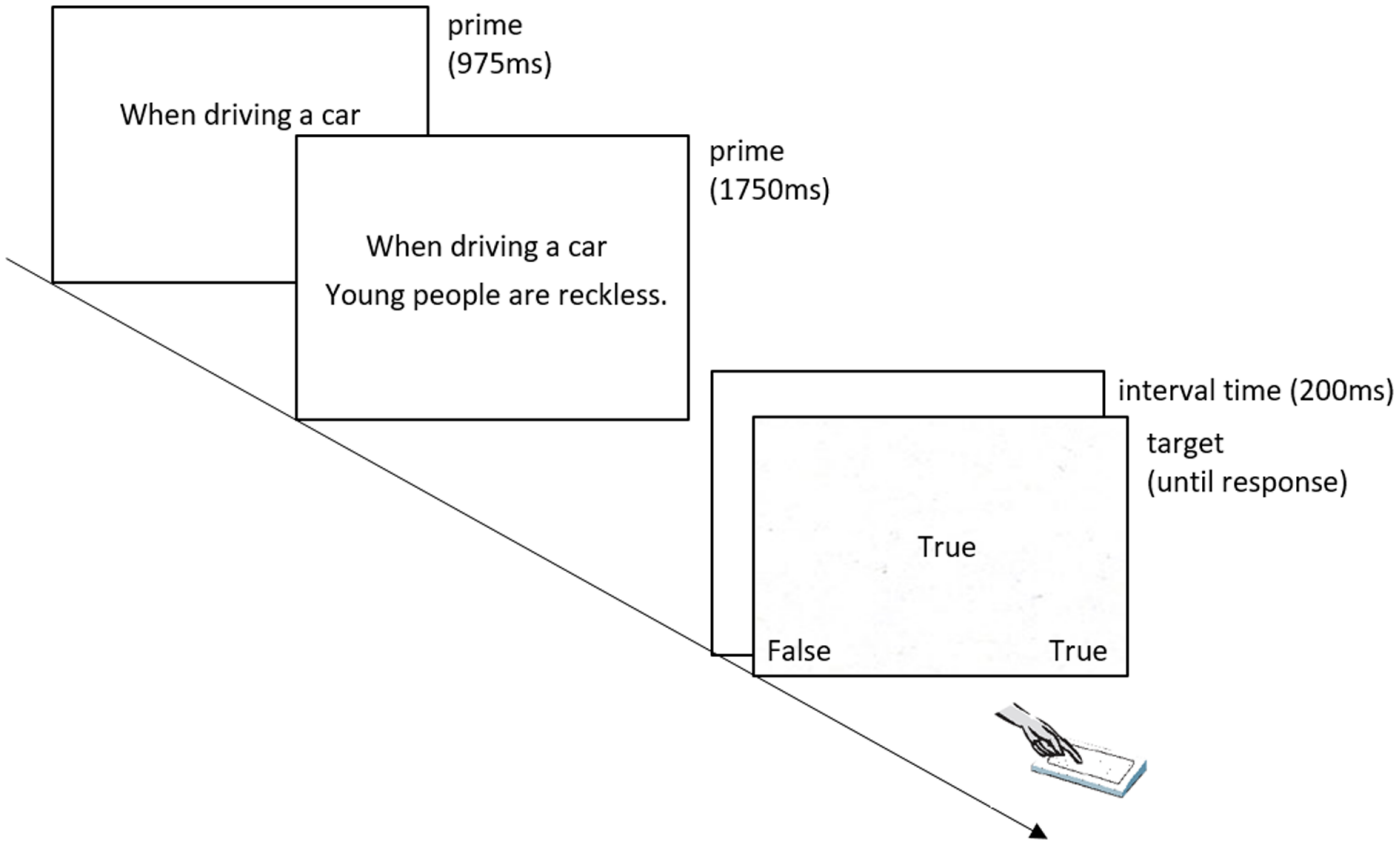
Presentation of an individual item in a PEP trial. Note that presentation time accounts for differences in word length.

The procedure of our study was based on Müller and Rothermund (2019), Exp. 1. The presentation of stimuli and recording of responses was controlled by Psychopy. The formal experiment started after a practice block which included 16 PEP trials. Each PEP trial had the following temporal sequence of events (see Figure 1): Firstly, the context phrase (e.g., “When driving a car”) was presented in the upper middle of the screen. Afterward, the context phrase stayed at the same position while the age category and attribute (e.g., “young people are reckless”) appeared in the lower middle of the screen. The rule for calculating the presentation duration of each word in the sentence was 150 plus 25 ms for each letter of the word. For example, the phrase “When driving a car” was presented separately for (150 ms × 4 words) + (25 ms × 15 letters) = 975 ms. For the category and attribute, an additional 600 ms was given. For example, “young people are reckless” was presented for (150 ms × 4 words) + (25 ms × 22 letters) + 600 ms = 1750 ms. After the prime presentation, the screen was blank for 200 ms. Then, the target word “True” or “False” was presented and participants were asked to press one of the two keys (“D” for “False” and “L” for “True”) to indicate whether the target was “True” or “False.” To ensure that statements were processed by participants with an evaluative mindset, in catch trials, the question “?True or False?” was presented instead of a target word and participants were asked to respond based on their personal evaluation of the previously shown statement. The target word or the question remained on the screen until a response was registered.

The eight stereotypic statements that are related to cautiousness or recklessness were assigned six times to one of the two target words, resulting in 96 PEP trials. The other 24 stereotypic statements that are not related to cautiousness or recklessness were assigned two times to one of the two target words, resulting in an additional 96 PEP trials. Each of the 32 stereotypic statements as well as another 32 non-stereotypic statements was assigned once to the question “?False or True?,” resulting in 64 catch trials. In total, there were 256 trials being presented in a random sequence.

After completing the PEP task, participants were asked to rate to which degree they agree with each of the 32 stereotypic statements that had been used as sentence primes in the PEP trials (e.g., “when driving a car, young people are reckless”). Ratings were given on a Likert scale ranging from 1 (totally disagree) to 7 (totally agree).

At the end, participants were asked how cautious vs. fast they responded during the response time task, on a Likert scale ranging from 1 (as cautious as possible) to 7 (as fast as possible). The self-reported cautiousness was recorded by subtracting the original value from 8 so that larger value of the self-reported cautiousness indicated more cautious responding in the PEP task.

## Results

### Data treatment

Erroneous responses (3.51%) and outlier values that were slower than 2,500 ms or faster than 150 ms (2.42%) were excluded from the analyses. Reaction times exceeding the third quartile of an individual’s respective reaction time distribution by more than three interquartile ranges (extreme outliers according to Tukey, 1977, 1.58%) were also removed. Two young and two older participants had to be excluded from data analyses for extremely high or low values of the PEP effects for sentences assessing the endorsement of the reckless or cautious stereotypes, with effect scores being more than three interquartile ranges above the 75th percentile or less than three interquartile ranges below the 25th percentile of the distribution in the respective age group (Tukey, 1977). The final sample sizes were 89 young (*M_age_* = 25.57 years, *SD* = 3.62) and 125 older (*M_age_* = 71.74 years, *SD* = 4.26) participants.

### Endorsement of age stereotypes

Mean reaction times for each of the eight conditions of the 2 (Category: matching vs. mismatching) × 2 (Context: relevant vs. irrelevant) × 2 (Required response: true vs. false) design were computed across all the eight stereotypic attributes for each young and old participant. We further calculated the endorsement effects of the four types of stereotypic statements varied in the dimension of Category and Context by subtracting mean reaction times for “true” responses from mean reaction times for “false” responses after the same statement, with larger scores indicating stronger endorsement.

**Figure 2:**
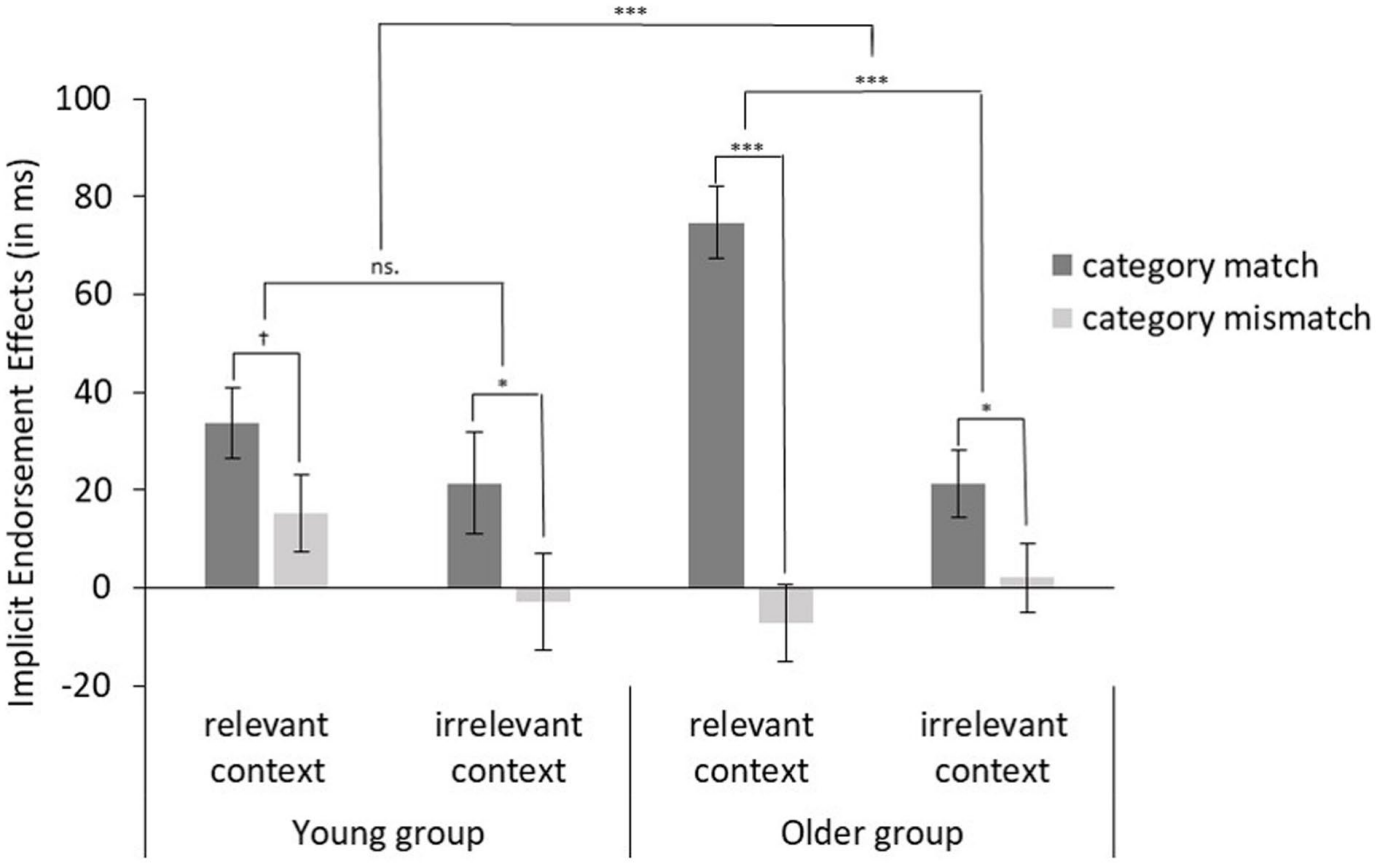
Implicit endorsement effects (error bars indicate standard errors) in the PEP depending on the category and context information in the statement and age group. ^†^*p* < 0.1, ^*^p < 0.05, and ^***^p < 0.001.

The endorsement effects for each of the four conditions were entered in a 2 (Category: matching vs. mismatching) × 2 (Context: relevant vs. irrelevant) × 2 (Age group: young vs. old) repeated ANOVA. As illustrated in Figure 2, we found a main effect of Category with stronger endorsement effects for the matching than for the mismatching category condition, *F*(1, 212) = 48.26, *p* < 0.001, partial ƞ^2^ = 0.19, and a main effect of Context with stronger endorsement effects for the relevant than for the irrelevant context condition, *F*(1, 212) = 14.59, *p* < 0.001, partial ƞ^2^ = 0.06. These main effects were qualified by a significant two-way interaction between Category and Context, *F*(1, 212) = 7.24, *p* = 0.008, partial ƞ^2^ = 0.03, indicating that in general, there was stronger implicit endorsement of age stereotypical beliefs about matching categories for relevant compared to irrelevant contexts. Unexpectedly, further simple effect analysis also showed endorsement effects for irrelevant context condition. Regarding age group differences, we found a three-way interaction of Category × Context × Age Group, *F*(1, 212) = 14.57, *p* < 0.001, partial ƞ^2^ = 0.06, suggesting that compared to young participants, older participants endorsed the stereotypic beliefs more strongly than younger participants.

**Figure 3:**
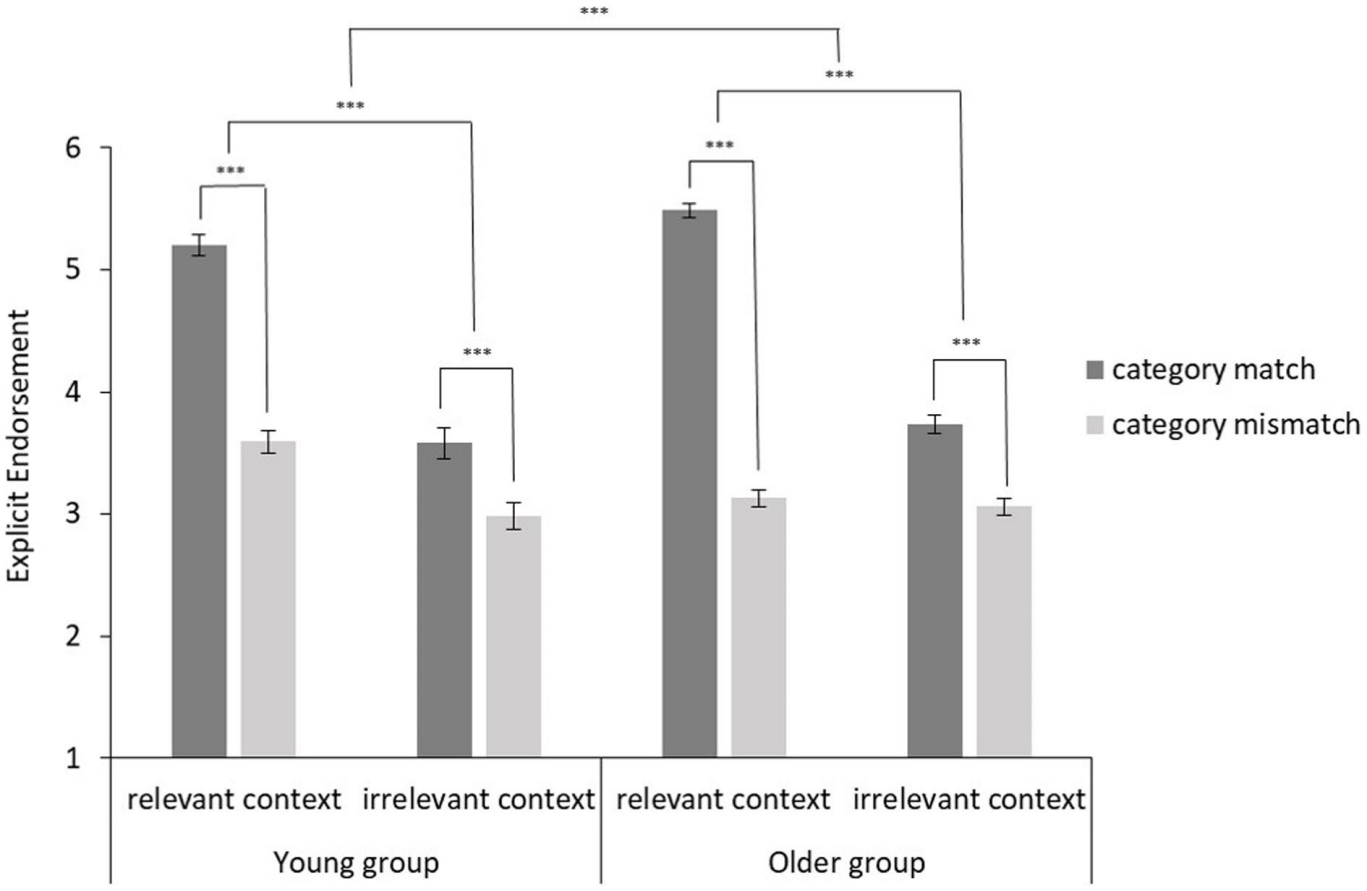
Explicit endorsement (error bars indicate standard errors) of age stereotypes depending on the category and context information in the statement and age group. ^***^p < 0.001.

A 2 (Category: matching vs. mismatching) × 2 (Context: relevant vs. irrelevant) × 2 (Age group: young vs. old) repeated ANOVA was also conducted with explicit ratings (see Figure 3). Similar to the implicit effects, we found a significant interaction between Category and Context, *F*(1, 212) = 349.01, *p* < 0.001, partial ƞ^2^ = 0.62, indicating that individuals endorsed the explicit age stereotypical beliefs about matching categories more strongly for relevant than for irrelevant contexts. We also found a significant three-way interaction with the factor of Age group, suggesting stronger endorsement of explicit age stereotypes among older adults, *F*(1, 212) = 21.68, *p* < 0.001, partial ƞ^2^ = 0.09.

### Embodiment of cautiousness-related age stereotypes

The parameter *a* was computed for each participant based on the individual RT distributions of correct and erroneous responses in the PEP using Fast-DM (Voss and Voss, 2007). Larger values of the parameter *a* indicated more cautious responding in the PEP task. The parameter *a* was then correlated with PEP effects reflecting the strength of endorsement of in-group age stereotypes about young people being reckless and old people being cautious, respectively, in the relevant contexts. Similarly, differences in the explicit endorsement of in-group stereotypes of being reckless (for the young sample) and cautious (for the sample of older participants) were also used to predict individual differences in self-reported cautiousness during the PEP. None of the correlations passed the significance threshold (see Tables 1, 2).

**Table 1:** Correlations between implicit and explicit endorsement of recklessness-related stereotypes in the relevant context and cautious behavior for the young group.

	*M*	*SD*	PEP effects	Explicit endorsement	Parameter *a*	Self-reported cautiousness
PEP effects	6.87	118.36	1	−0.06	0.12	0.02
Explicit endorsement	4.37	1.70		1	0.07	0.07
Parameter *a*	1.54	0.59			1	−0.00
Self-reported cautiousness	4.75	1.93				1

**Table 2:** Correlations between implicit and explicit endorsement of cautiousness-related stereotypes in the relevant context and cautious behavior for the older group.

	*M*	*SD*	PEP effects	Explicit endorsement	Parameter *a*	Self-reported cautiousness
PEP effects	89.04	129.80	1	0.17	0.03	0.11
Explicit endorsement	6.07	1.43		1	−0.04	0.06
Parameter *a*	1.71	0.50			1	0.03
Self-reported cautiousness	5.30	1.51				1

## Discussion

### Endorsement of age stereotypes and age difference

For both implicit and explicit measures, we found strong endorsement of age stereotypical beliefs among young and older adults when matching categories and attributes were combined with relevant contexts. In accordance with previous studies (Casper et al., 2011; Kornadt and Rothermund, 2011, 2015; Huang and Rothermund, 2022), our findings provide additional evidence for the context-dependent nature of the representation of age stereotypes. One finding that is inconsistent with the previous findings is that we also found small endorsement effects of age stereotypic beliefs in the unrelated contexts. These effects in irrelevant context condition were unusual and might be due to the specific small set of stereotypic traits being included in our study, which comprised only half of the stereotypic traits that had been used in the previous studies.

Beyond those previous studies mainly focusing on either young or older individuals, our study included both young and older samples, thus allowing us to compare the strength of endorsement of age stereotypes between the two age groups. Our results suggest that older individuals endorse age stereotypical beliefs to a stronger degree than young adults do, and this pattern was apparent for both explicit and implicit age stereotypes (for a similar pattern of findings regarding the implicit and explicit endorsement of prescriptive age stereotypes, see de Paula Couto et al., 2022b). The stronger tendency of endorsing age stereotypes among older adults may reflect (1) a longer history of stereotype-related experiences (relating to both age groups), which might confirm and strengthen that the age stereotypes were acquired earlier in life (for a related discussion, see de Paula Couto et al., 2022b); and (2) a chronic suppression failure to inhibit age stereotypic thoughts due to inhibitory deficits (von Hippel et al., 2000; Radvansky et al., 2010).

### Embodiment of cautiousness-related stereotypes

Unexpectedly, the implicit endorsement of cautiousness-related age stereotypes was found to be unable to predict young or older adults’ cautious vs. fast behavior in a speeded response time task. Apparently, this null finding cannot be attributed to an inadequate assessment of age stereotypes given that we employed a well-established paradigm to capture implicit stereotypical beliefs by introducing the contextual and relational information in the measurement, as was also evident by highly reliable stereotype endorsement effects for this measure in our study. With regard to the assessment of cautious behavior, we used an indirect criterion of speed-accuracy tradeoffs, namely the parameter *a* of the diffusion model which has been validated as a reliable indicator of the participants’ cautiousness (Voss et al., 2004). Despite the reliable assessment of predictor and outcome, we still found no evidence for an embodiment of implicit age stereotypes. In the following paragraph, we would discuss different explanations for the null findings as well as possible implications from the current study.

Former studies revealed greater impact of age stereotypes or views on aging on the related behaviors when the stereotype content corresponds to the outcome domain (Levy and Leifheit-Limson, 2009; Kornadt and Rothermund, 2015; Kornadt et al., 2020). Meeting this domain-specific rule by predicting cautious behavior with the cautiousness-related age stereotypes, however, we still could not find any prediction effects. A possible explanation for the null findings is that age stereotypes may exert their influence not only in a domain-specific manner but also in a context-specific manner. In the current study, the context that was provided during the assessment of age stereotypical beliefs in the PEP and rating tasks (“when driving a car”; “when protecting themselves against the Corona virus”) was different from the context in which the actual behavior was observed (i.e., during the PEP task). It is possible that the cautiousness-related stereotypical beliefs being triggered in specific contexts are independent of the cautious behavior being assessed in different contexts and the lack of match between the contexts that were used to assess the stereotypes and the stereotypic behavior may lead to the null finding. Thus one practical implication for future study that attempts to predict behaviors with implicit age stereotypes is that the principle of correspondence should be optimized to a maximal extent by using the same context for assessing stereotypical beliefs and behavioral outcomes (Ajzen and Fishbein, 1977).

Another explanation for the null effects of stereotype embodiment in our study is that aging stereotypes may only become internalized into one’s self-concept and exert their influence when the older individuals more strongly identify themselves as old people (Levy, 2003; Kornadt and Rothermund, 2012). It has been well demonstrated by many studies that the effects of age-stereotype primes rely on older participants’ identities as old people (e.g., Levy, 1996; Levy et al., 2002; Hess et al., 2004) and with younger participants, the age-stereotype-congruency effects could not be found or replicated (Bargh et al., 1996; Doyen et al., 2012). Considering that a large number of studies have shown that older adults who identify less strongly with their age group are less susceptible to the detrimental effects of negative age stereotypes (O'Brien and Hummert, 2006; Kang and Chasteen, 2009; Weiss et al., 2013; Armenta et al., 2018), it is thus possible that the stereotype embodiment effects could become stronger among older participants who have higher age identification with their peers. For the future research, it is worthwhile to assess age identification or subjective age and examine their potential moderating effects on the relation between one’s endorsement of implicit age stereotypes and the related behavioral outcomes.

### Limitation and future outlook

As a first attempt to examine the embodiment of implicit age stereotypes in the domain of spontaneous cautious behavior, our findings are open to different interpretations. The following limitations should thus be noticed when interpreting our findings. First of all, a large number of studies using a subliminal priming technique have demonstrated that the priming effects of age stereotype on the older individuals’ performance (e.g., physical activity; Meisner et al., 2013, memory; Levy, 1996, or will to live; Levy et al., 2000) are produced through the implicit aging self-stereotypes being activated by exposure to age-stereotype primes (Levy, 1996). The lack of the assessment of aging self-stereotypes in our study as well as in former studies showing no predictive validity of implicit measures of age stereotypes (e.g., Lindner, 2011; Ruiz et al., 2015) makes it difficult to differentiate the internalization process from the embodiment process of implicit age stereotypes and to make theoretical implication regarding the processes of self-stereotyping, and embodiment in the realm of implicit age stereotypes. To accurately answer whether and to which degree the implicit age stereotypical beliefs become assimilated into one’s self-views and further exert their impact on one’s judgment or behavior, future studies should assess not only implicit age stereotypes and the behavioral outcomes but also the related self-views. Secondly, the current study investigated the embodiment of implicit age stereotypes by focusing on the impact of cautiousness-related age stereotypes on spontaneous cautious behavior. To expand our understanding of the embodiment process of implicit age stereotypes as well as its operating conditions, future studies should examine different subtypes of explicit and implicit age stereotypes and their influence on the related functions or behavioral outcomes. For example, referring to the stereotype content model (SCM, Cuddy et al., 2008), it would be very interesting to examine whether older (or young) individuals endorse and internalize the implicit age stereotypes that older people are warm but incompetent (or younger people are competent but less warm).

### Summary

To conclude, we found strong endorsement of implicit age stereotypes in young and older adults but no prediction of implicit age stereotypes on cautious behavior, which allows us to put forward the following speculations that (1) age stereotypes may only predict the age-related behavior when they are both assessed in the same contexts; and (2) age stereotypes may only become part of one’s self-concept *via* internalization process and exert their influence when the individuals identify with their own age group. Given that a single study is seldom sufficient to yield definite conclusions, more research is needed that tests for an embodiment of implicit stereotypes with stereotypes and behaviors that refer to more similar contexts, different domains of functioning (i.e., warmth vs. competence), to examine the potential moderating role of age identification or subjective age in the self-stereotyping process, and also to differentiate between (implicitly assessed) general age stereotypes and personalized views of aging as predictors of embodiment. In our view, such follow-up studies still have a lot of potential to demonstrate internalization effects by addressing the limitations of the experimental setup and operationalization of the current study.

## Data availability statement

The datasets presented in this study can be found in online repositories. The names of the repository/repositories and accession number (s) can be found in the article/supplementary material.

## Ethics statement

The studies involving human participants were reviewed and approved by the study has been reviewed and approved by Friedrich-Schiller University Jena, FSV 19/37. The patients/participants provided their written informed consent to participate in this study.

## Author contributions

TH: conceptualization, methodology, data curation and analysis, writing, and editing. KR: conceptualization, methodology, writing, and editing. All authors contributed to the article and approved the submitted version.

## Funding

This study was funded by a grant that was awarded as part of the program “Preregistration in Psychology” of the Leibniz Institute “Zentrum für Psychologische Information und Dokumentation (ZPID)” to TH and KR. We acknowledge support by the German Research Foundation Projekt-Nr. 512648189 and the Open Access Publication Fund of the Thueringer Universitaets- und Landesbibliothek Jena.

## Conflict of interest

The authors declare that the research was conducted in the absence of any commercial or financial relationships that could be construed as a potential conflict of interest.

## Publisher’s note

All claims expressed in this article are solely those of the authors and do not necessarily represent those of their affiliated organizations, or those of the publisher, the editors and the reviewers. Any product that may be evaluated in this article, or claim that may be made by its manufacturer, is not guaranteed or endorsed by the publisher.

## Footnotes

^1^Due to a technical problem during the data collection, the final sample size of older participants (N = 127) was larger than we preregistered (N = 90). The analyses only including the first 90 older participants who participated in the study revealed the same pattern as the analyses including the whole sample of 127 older participants. The results being presented in the current study are based on the complete sample of young and older participants.

^2^ https://doi.org/10.23668/psycharchives.5376

^3^ https://doi.org/10.23668/psycharchives.7975
